# Impact of UV-Irradiated Mesoporous Titania Nanoparticles (mTiNPs) on Key Onco- and Tumor Suppressor microRNAs of PC3 Prostate Cancer Cells

**DOI:** 10.3390/genes16020148

**Published:** 2025-01-25

**Authors:** Andrea Méndez-García, Luis Alberto Bravo-Vázquez, Padmavati Sahare, Sujay Paul

**Affiliations:** 1 Tecnologico de Monterrey, School of Engineering and Sciences, Campus Queretaro, Av. Epigmenio González No. 500 Fracc. San Pablo, Querétaro 76130, Mexico; 2 Tecnologico de Monterrey, Institute of Advanced Materials for Sustainable Manufacturing, Epigmenio González No. 500 Fracc. San Pablo, Querétaro 76130, Mexico

**Keywords:** mesoporous titania nanoparticles, prostate cancer, microRNAs, UV-irradiation, nano-therapeutics

## Abstract

Background: Mesoporous titanium dioxide nanoparticles (mTiNPs) are known for their chemical stability, non-toxicity, antimicrobial and anticancer effects, as well as for their photocatalytic properties. When this material is subjected to UV radiation, its electronic structure shifts, and during that process, reactive oxygen species are generated, which in turn exert apoptotic events on the cancer cells. Objectives: We evaluated the cytotoxic effects of UV-irradiated mTiNPs on prostate cancer (PCa) cell line PC3 with the aim of demonstrating that the interaction between UV-light and mTiNPs positively impacts the nanomaterial’s cytotoxic efficiency. Moreover, we assessed the differential expression of key oncomiRs and tumor suppressor (TS) miRNAs, as well as their associated target genes, in cells undergoing this treatment. Methods: PBS-suspended mTiNPs exposed to 290 nm UV light were added at different concentrations to PC3 cells. Cell viability was determined after 24 h with a crystal violet assay. Then, the obtained IC_50_ concentration of UV-nanomaterial was applied to a new PC3 cell culture, and the expression of a set of miRNAs and selected target genes was evaluated via qRT-PCR. Results: The cells exposed to photo-activated mTiNPs required 4.38 times less concentration of the nanomaterial than the group exposed to non-irradiated mTiNPs to achieve the half-maximal inhibition, demonstrating an improved cytotoxic performance of the UV-irradiated mTiNPs. Moreover, the expression of miR-18a-5p, miR-21-5p, and miR-221-5p was downregulated after the application of UV-mTiNPs, while TS miR-200a-5p and miR-200b-5p displayed an upregulated expression. Among the miRNA target genes, *PTEN* was found to be upregulated after the treatment, while *BCL-2* and *TP53* were underexpressed. Conclusions: Our cytotoxic outcomes coincided with previous reports performed in other cancer cell lines, strongly suggesting UV-irradiated mTiNPs as a promising nano-therapeutic approach against PCa. On the other hand, to the best of our knowledge, this is the first report exploring the impact of UV-irradiated mTiNPs on key onco- and TS microRNAs in PCa cells.

## 1. Introduction

Prostate cancer (PCa) ranks as the second most recurrent malignancy in men, closely following lung cancer. Additionally, it has been recognized as the fifth most significant cause of death on the global scale [1,2,3]. The main common factors that trigger the development of this disease include advanced age, multiple genetic variations (e.g., mutations in *BRCA*, *HOXB13*, *PTEN*, *RAS*, and/or *CHEK2* genes), hereditary predisposition, obesity, smoking, selenium deficiency, and frequent dairy ingestion [4,5,6,7,8]. Remarkably, at least 1,466,680 new PCa cases, along with 396,792 related deaths, were registered worldwide in 2022, according to the GLOBOCAN 2022 cancer estimates published in 2024 [9]. In fact, the prevalence of PCa increases dramatically with age; for instance, men over 80 years of age are diagnosed 40 times more often compared to those under 50 years old [5].

MicroRNAs (miRNAs) are small (18–24 nt) non-coding RNAs (ncRNAs) that regulate gene expression at the post-transcriptional level. These tiny single-stranded molecules bind to a complementary sequence located at the 3′-untranslated region (UTR) of their target messenger RNA (mRNA), leading to its degradation or a physical translational blockage, ultimately inhibiting its expression [10,11,12]. Due to their importance in the regulation of a number of biological processes, miRNAs can function as either oncomiRs or tumor suppressors (TS), depending on the target genes they regulate. OncomiRs are miRNAs that promote cancer progression by downregulating TS genes, thereby facilitating processes such as cell proliferation and apoptosis inhibition. Conversely, TS miRNAs impede cancer development by targeting oncogenes, preventing uncontrolled cell growth and promoting programmed cell death [13,14,15,16].

It is worth noting that multiple novel therapeutic approaches have been developed to overcome the shortcomings of conventional chemotherapy against cancer, such as limited drug bioavailability and off-target toxicity [17,18]. One of these alternatives has been centered on nanotechnology. For instance, it has been demonstrated that lipid nanomaterials facilitate the delivery of RNA-based drugs into cancer cells [19], while other nanotechnological approaches can be applied to trigger DNA damage, thereby mediating cancer cell death [20]. Particularly, metal-based nanoparticles (NPs) are able to induce a redox imbalance inside the malignant cells, ultimately triggering apoptosis [21]. Titanium dioxide (TiO_2_) NPs (TiNPs) have been recognized as outstanding metallic nanomaterials due to their chemical stability, non-toxicity, antimicrobial effects, photo-activation properties, biocompatibility, and anticancer activity [22,23]. The most common crystallographic structures of TiNPs are rutile, brookite, and anatase, the latter being the most frequently applied in cancer therapeutics [24,25,26].

The photocatalytic property of TiNPs has been its most outstanding advantage when employing them for therapeutic purposes. The elevated refractive index of this material grants it the potential to absorb UV photons, which induces the disintegration of water molecules and, hence, the release of -OH radicals, superoxide anions, and singlet oxygen, activating oxidative stress-induced apoptosis [26,27]. Even though studies that support an active photocatalytic effect of amorphous TiNPs are scarce, the ones available suggest that this process can be triggered spontaneously, highly depending on the experimental conditions [28,29,30].

Various in vitro and in vivo cancer models have demonstrated the activation of apoptotic and necrotic pathways after treatments with TiNPs as a response to the oxidative stress and DNA damage exerted by these NPs [22,25]. Markowska-Szczupak [31] noticed increased mortality of breast adenocarcinoma MCF7 cells under UV-A/vis-activated TiO_2_, in contrast to cells treated with non-irradiated material. Later, Balachandran et al. [32] used UV-irradiated anatase-configured TiNPs as a treatment against the A549 lung cancer cell line, and they achieved an outstanding result, as 85% of the cells were decomposed in a 4 h interval. Likewise, Bilkan et al. [27] evaluated the combined effect of UV radiation and TiNPs on skin and breast cancer cell lines, and they found that a considerable number of cells went through an apoptotic process when the combined UV-A + TiO_2_ treatment was applied. On the other hand, another group reported the molecular mechanisms by which TiNPs are capable of inhibiting the growth of hepatocarcinoma cell line HepG2 [33]. They demonstrated that this nanotreatment triggers cancer cell death by arresting the cell cycle in the G1 phase and inducing oxidative stress. Concretely, they showed that endoplasmic reticulum membrane-bound transcription factor (*ATF6*) and protein kinase RNA-like endoplasmic reticulum kinase (*PERK*) were upregulated when high reactive oxygen species (ROS) levels were detected in liver cancer cells treated with UV-A-stimulated TiO_2_ [33]. The activation of the ATF6/PERK molecular pathway led to the overexpression of C/EBP homologous protein (*CHOP*), which triggered the caspase cascade and ultimately executed the endoplasmic reticulum stress-induced apoptosis [33]. Maddah et al. [34] also showed that a TiNP treatment applied to the HCT166 colon cancer cell line substantially reduced the survival of the cancerous cells by increasing ROS production and decreasing the overall activity of antioxidant enzymes.

Even though there are extensive reports on the potential mechanisms by which TiNPs-based treatments induce oxidative stress and apoptotic responses, their impact on the miRNA expression in cancer cells is still elusive. Indeed, only a couple of works showed the effect of TiNPs on a few onco- and TS miRNAs of lung and colon cancer. For example, it was observed that miR-21, a key biomarker for carcinogenesis [35], was persistently downregulated in A549 cell cultures upon TiNP treatment [36]. Its limited expression facilitated cell cycle arrest, enhanced chemosensitivity, and allowed the reduction of tumor proliferation, mitigation, and invasion. More importantly, this oncomiR, along with TS-miRNA miR-155, was proven to post-transcriptionally regulate the autophagic process associated with ER and mitochondrial dysfunction [36]. Likewise, an assay performed with HTC116 colon cancer cell line showed that, upon exposure to TiNPs, TS-miRNAs miR-378e and miR-199b-3p had upregulated by at least a 2-fold magnitude and that the oncomiR miR-378i was expressed at least 6 times below the normal levels [37]. Interestingly, the expression of all these ncRNAs was directed towards preventing cancer progression [38,39,40,41]. However, the impact of UV-activated mesoporous TiNPs (mTiNPs) on crucial onco- and TS-miRNAs has been, until now, remained completely unexplored in any cancer. Under this premise, the purpose of this work was to evaluate the cytotoxic effects of UV-irradiated mTiNPs on the PC3 cell line and their impact on key onco- and TS-miRNAs.

## 2. Materials and Methods

### 2.1. mTiNPs Synthesis and Characterization

mTiNPs were synthesized via sol-gel and soft hydrothermal methods, as outlined by Borjas-Garcia et al. [42], with minor modifications. The synthesis was carried out in three distinct phases. The initial phase entailed the preparation of a stock Ti-solution by vigorous mixing of 0.25 mol of triethanolamine (TEAOH) and 0.125 mol of titanium butoxide for 24 h. In the second phase, 3.19 g of the previously produced stock Ti-solution was combined with 0.91 g of hexadecyltrimethylammonium bromide (CTAB) in 10 g of distilled water. In the final phase, 1.8 mL of a 0.88% (*w*/*w*) NaOH solution was added dropwise to the previously prepared Ti-CTAB solution while stirring continuously, resulting in the formation of a gel. The obtained material was heated at 40 °C while stirring for one day with the aim of evaporating the excess humidity; then, the remaining material was stored in a closed glass flask at 80 °C for 24 h, washed with 100 mL of distilled water, and centrifuged thrice at 12,000 rpm. The solid phase was dried at 80 °C for one day and immediately calcined at 560 °C for 1 h to eliminate any residual organic template. After that, 1 g of the material was dispersed into 1 L of pure ethanol and aliquoted in fractions of 50 mL. Each tube was ultrasonicated in a range of 38–41 V for 38 cycles of 2.5 min each, leaving a 30 s interval in between. All samples were centrifuged at 10,000 rpm for 10 min at room temperature, the supernatant was discarded, and the solid material was left to air-dry.

The surface morphology of the samples was carried out using a Hitachi SU8230 cold field emission scanning electron microscope (CFE-SEM, Hitachi, Tokyo, Japan) working at a low voltage of acceleration (3 keV). The particle size of the synthesized mTiNPs was acquired by dynamic light scattering (DLS) using the SZ-100 series instrument (Horiba Scientific, Kyoto, Japan). Specific surface area, pore size, and pore volume were determined using the Quantachrome TouchWin instrument (Quantachrome Instruments, Boynton Beach, FL, USA). The sample was outgassed in a vacuum at 150 °C for 4 h before measurement. The specific surface area was measured by N_2_ adsorption/desorption at 77 K using the Brunauer–Emmett–Teller (BET) method, and the pore size distribution from the desorption branch of the isotherm was calculated by the Barrett–Joyner–Halenda method. Finally, the organic chemical structure of the dry nanomaterial was evaluated using Fourier-transform infrared spectroscopy (FTIR) with a Spectrum Two FT-IR Spectrometer (Perkin Elmer, Waltham, MA, USA), set to a wavelength range of 500 to 4000 cm^−1^.

### 2.2. Cell Culture

Human cell line PC3, a PCa cell line widely used in the field of nanotherapeutic research [43,44,45,46,47], and Human Embryonic Kidney (HEK) 293 cells were obtained from the American Type Culture Collection (ATCC). The cells were grown in Dulbecco’s Modified Eagle Medium (DMEM) containing high glucose, supplemented with 10% Fetal Bovine Serum (FBS), and 1% penicillin–streptomycin antibiotic. To achieve the desired cell confluence, all cultures were maintained in a 5% CO_2_ humidified incubator at 37 °C.

### 2.3. In Vitro Cytotoxicity Assay

Although the MTT assay is the most common technique to evaluate the cytotoxic effects a variety of materials have on cells, there have been reports demonstrating photocatalytic interactions between mTiNPs and the MTT [48]. Therefore, in this study, we performed a crystal violet (CV) assay with PC3 and HEK 293 cells to assess their viability. PC3 was first cultured in high-glucose DMEM until 80% confluency was achieved. Then, 960,000 cells were evenly distributed and incubated in a 96-well plate (10,000 cells per well) for 24 h at 37 °C and 5% CO_2_. A stock solution of 73.3 nm mTiNPs suspended in PBS (480 µg mL^−1^) was UV-C-irradiated at 290 nm using a 30 W UV lamp for 2 h and immediately added to the cells at concentrations of 1, 10, 30, 60, 90, 120, 150, 180, and 210 µg/mL, respectively. The same concentrations of non-UV-irradiated mTiNPs were used in a second experimental group. The positive control consisted of cells without serum supplementation, a condition that did not allow enhanced growth, while the negative control consisted of cells in media enriched with serum, ensuring correct cell development. The treated and untreated cells were incubated at 37 °C and 5% CO_2_ for 24 h. After incubation, the medium was removed, and the wells were washed with PBS. After that, the cells were fixed with 4% paraformaldehyde for 15 min and washed with PBS. Finally, 0.5% (*w*/*v*) crystal violet solution was added to each well and incubated for 20 min at 25 °C. The plates were washed with copious water to remove the unbound dye. After drying the plates, methanol was added, and the absorbance was determined spectrophotometrically. Similarly, cell viability assessment of UV-irradiated mTiNPs was conducted on HEK 293 cells to assess their impact on healthy cells. The IC_50_ analyses for the non-UV- and UV-irradiated mTiNPs samples were performed using a linear expression. Specifically, dose-response data were plotted, and a linear regression model was applied to the linear portion of the curve. The experiment was performed using three biological triplicates, and each concentration was also carried out in triplicates.

### 2.4. RNA Extraction and Expression Evaluation by qPCR

Due to the significantly enhanced cytotoxic effects of UV-irradiated mTiNPs on PC3 cells compared to the non-irradiated nanomaterial, miRNA analyses were focused exclusively on the cells with UV-irradiated mTiNPs treatment. PC3 cells were first seeded into T25 flasks and incubated until 80% confluency was achieved. The cells were then treated with 48.96 µg/mL of UV-irradiated mTiNPs that had a size of 73.3 nm. The optimal concentration was calculated based on the IC_50_ values from the previously performed cytotoxic assays. The control and treated cells were left for incubation at 37 °C and 5% CO_2_ for 24 h. The total RNA from the treated and untreated cells was isolated and purified using the miRNeasy Mini Kit (Qiagen, Hilden, Germany) following the manufacturer’s protocol. Then, the quality and concentration parameters of the extracted RNA were evaluated using the NanoDrop One spectrophotometer (Thermo Scientific, Wilmington, NC, USA). Subsequently, 1 μg of the total RNA was polyadenylated and reverse transcribed using the SMART MMLV Reverse Transcriptase contained in the mRQ Enzyme Mix of the Mir-X miRNA First-Strand Synthesis Kit (Takara, Tokyo, Japan).

The qPCR analyses were performed in the Step One Real-Time PCR System (Applied Biosystems, Carlsbad, CA, USA) using the reagents of the Mir-X miRNA qRT-PCR SYBR Kit (Takara, Tokyo, Japan). The complete sequence of each selected miRNA was used as the forward primer, while the mRQ 3′ primer provided by the abovementioned kit functioned as the reverse primer. Additionally, forward and reverse primers, specific for PCa-related genes, were used to analyze target gene expressions. The primers used in the qPCR experiments are listed in Table 1. The reactions were prepared in a final volume of 12.5 μL containing 1× SYBR Advantage Premix, 1× ROX dye, 0.2 µM of both forward and reverse primers, and 1 µL of the first-strand cDNA. A total of six miRNAs with previously documented oncogenic or TS functions [49,50,51,52] were selected for qRT-PCR: miR-16-5p, miR-18a-5p, miR-21-5p, miR-200a-5p, miR-200b-5p, and miR-221-5p. In addition, the relative expression levels of B-cell lymphoma-2 (*BCL-2*), tumor protein p53 (*TP53*), and phosphatase and tensin homolog (*PTEN*), target genes associated with these cancer-related miRNAs, were also analyzed. The conditions of the qPCR reactions were set considering a starting denaturation stage at 95 °C for 10 s, 45 cycles of denaturation at 95 °C for 5 s, annealing at 55 °C for 20 s, and extension at 72 °C for 20 s, ending with a melting curve of 95 °C for 1 min, 55 °C for 30 s, and 95 °C for 30 s. All the qPCR reactions were carried out in duplicates with at least two technical replicates for both control and treated cells [53,54,55]. Finally, the relative fold changes in miRNA and gene expression were calculated following the comparative Ct method, also referred to as the delta–delta Ct method (2^−ΔΔCT^), using the U6 as the endogenous normalization control.

**Figure 1:**
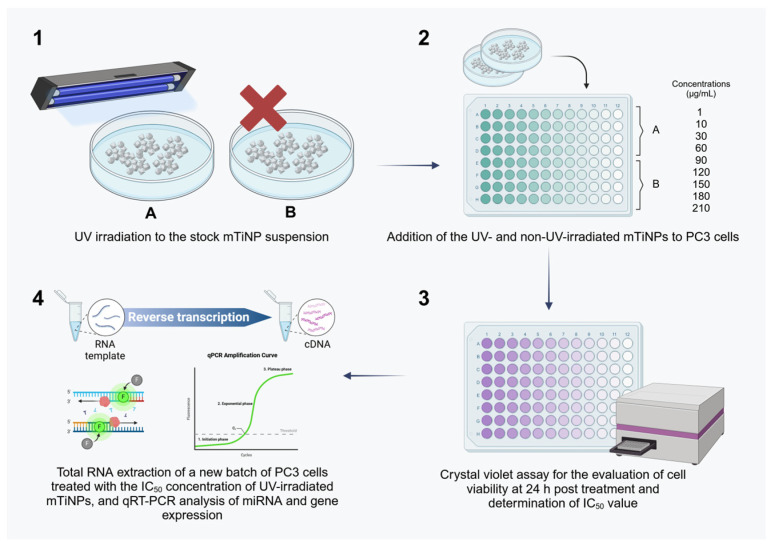
The methodology was followed during the application of mTiNP treatments to PC3 cells and in the subsequent evaluation of miRNA/target gene expression. (**A**) UV-irradiated mTiNPs and (**B**) non-UV-irradiated mTiNPs (created with a licensed version of Biorender).

A schematic overview of the workflow followed for the application of the mTiNP treatments applied to PC3 cells and the subsequent evaluation of miRNA/gene expression are depicted in Figure 1.

### 2.5. Statistical Analysis

The Student’s *t*-test was applied to evaluate the statistical significance between the groups of the biological replicates, with a *p*-value < 0.05 being considered statistically significant. The results are presented in the graphs as the mean value ± the standard error of the biological replicates.

## 3. Results and Discussion

### 3.1. Characterization of mTiNPs

**Figure 2:**
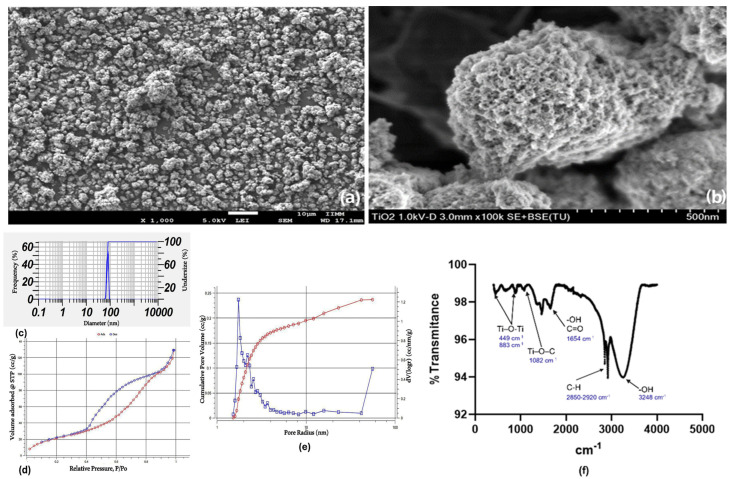
Results of the characterization of the TiNPs: (**a**,**b**) SEM images of synthesized mTiNPs; (**c**) particle size distribution of mTiNPs; (**d**) nitrogen adsorption/desorption isotherms; (**e**) pore size distribution of the mTiNPs; (**f**) FTIR analysis of dry mTiNPs.

The microstructure of the synthesized mTiNPs was explored by SEM, observing the expected regular spherical particles with a clearly visible porous network (Figure 2a,b). The particle size distributions of the synthesized mTiNPs were analyzed using DLS, which confirmed a size range between 64 nm to 83 nm, with a prominent peak at 73.3 nm (mean size) (Figure 2c). In addition, Figure 2d depicts the N_2_ adsorption/desorption isotherms of mTiNPs. The BET-specific surface area of the mTiNPs was found to be 171.446 m^2^/g, with a pore diameter of 3.46 nm and a pore volume of 0.236 cc/g, respectively. Additionally, our mTiNPs sample showed a significant hysteresis loop at relatively high pressure, which was attributed to capillary condensation related to large pore channels, implying the existence of mesopores type IV isotherms according to the IUPAC classification [56]. The corresponding BJH pore size distributions calculated from the desorption data of the isotherms are shown in Figure 2e.

NPs with smaller sizes (<100 nm), together with an optimized pore network, allow the material to exhibit greater reactivity compared to larger particles, resulting in better cytotoxicity effects and improved cost-effectiveness. The main reason why NPs are characterized by extensive surface area is based on the geometric principle of surface-to-radius proportion. This mathematic postulate states that generating more particles with smaller radii from one original volume through extensive physical divisions allows each particle to contribute cumulatively to the overall surface area, building a larger space for chemical reactions [57].

The FTIR spectra in the range 4000–400 cm^−1^ are presented in Figure 2f. The discrete peaks between 440 cm^−1^ and 900 cm^−1^ are due to the presence of stretching Ti–O–Ti. Meanwhile, the band immediately after 1000 cm^−1^ is related to the Ti–O–C group. The peak at 1654 cm^−1^ is commonly assigned to the bending vibration of the -OH group and even the stretching of carboxylate groups (C=O), due to the presence of titanium isopropoxide and ethanol as precursors. Additionally, two sharp peaks around 2920 and 2850 cm^−1^ correspond to the stretching vibration of the aliphatic C-H bond. Finally, the peak observed between 3000 and 4000 cm^−1^ represents the stretching vibration of the hydroxyl group -OH, related to water moisture. The intensity of this band indicates the degree of surface hydroxylation, which is crucial for photocatalytic activity [58,59,60,61,62].

### 3.2. Cytotoxicity Assay of PC3 Cells Treated with UV and Non-UV-Irradiated mTiNPs and HEK 293 Treated with UV-Irradiated mTiNPs

**Figure 3:**
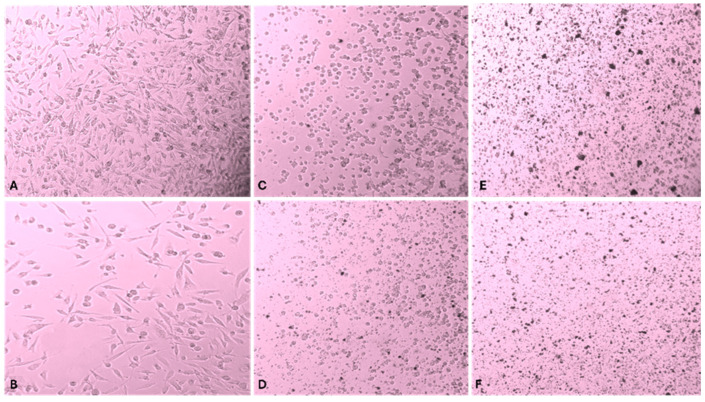
Microscopic images of PC3 cells at 40× magnification. (**A**) Negative Control; (**B**) Positive Control (without serum); (**C**) 10 µg/mL non-UV; (**D**) 10 µg/mL UV; (**E**) 120 µg/mL non-UV; (**F**) 120 µg/mL UV.

The morphology of baseline PC3 cells was examined under the microscope at 40X magnification, as well as those exposed to the lowest (10 µg/mL) and highest (120 µg/mL) therapeutic concentrations. As expected, the appearance of the positive control (Figure 3A) showed spindle-shaped cells with optimal adherence to the flask and adequate cell-to-cell contact, which favored rapid growth and cell division. Additionally, the culture exhibited even and confluent distribution in a monolayer pattern. On the contrary, the negative control (Figure 3B), lacking serum supplementation, displayed a scattered distribution, minimal confluency, and limited growth. Cells treated with the 10 µg/mL concentration of mTiNPs (Figure 3C,D) exhibited a circular shape, a sign of a stress response, while the irregular borders indicated cell damage. The cells exposed to non-UV treatment (Figure 3C) showed a considerably larger size than those where UV-mTiNPs were added (Figure 3D). Finally, PC3 cells that were subjected to the highest concentration of UV-mTiNPs and mTiNPs (Figure 3E,F) had no discernible structure, indicating serious damage that led to apoptosis. The morphology of control and stressed cells was comparable to those treated with an isoalantolactone apoptotic agent [63].

**Figure 4:**
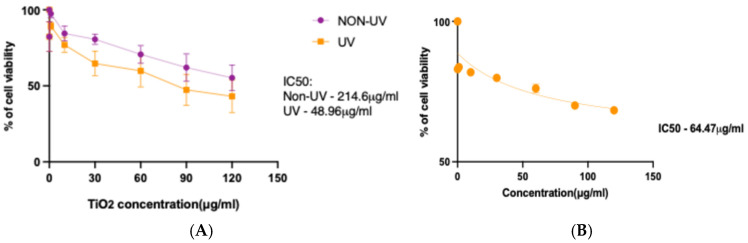
Viable PC3 cells (%) after 24 h exposure to different concentrations of UV irradiated mTiNPs (orange) and mTiNPs without photocatalytic treatment (purple) (**A**). The viability of HEK 293 to different concentrations of UV-irradiated mTiNPs is also shown in (**B**).

Cell viability was assessed 24 h after applying a series of increasing concentrations of both treatments. The highest inhibition achieved with non-UV TiNPs was 44.75% when employing a concentration of 120 µg/mL, while the inhibition percentage for UV TiNPs was 56.84% for that same concentration. Moreover, the mean inhibitory concentration (IC_50_) was calculated based on the viability tendency, revealing a delta of 165.64 µg/mL between the two treatments (Figure 4A). In this case, UV-TiNPs required a 4.38-fold lower concentration in comparison with the non-irradiated titania to achieve the same percentage of biological inhibition. The IC_50_ value of HEK 293 treated with UV-irradiated mTiNPs was found to be 64.47 µg/mL (Figure 4B).

In a similar assay, but performed on a chondrosarcoma SW 1353 cell line, it was found that TiNPs exerted cytotoxic effects in a dose-dependent matter. The authors encountered that the IC_50_ of non-irradiated TiNPs applied for 24 h was 211.3 ± 15.2 µg/mL [64], which is comparable to our results. Further, the IC_50_ of the same treatment, but applied to the colorectal adenocarcinoma HT29 cell line, was 198.849 μg/mL [65], similar to our findings. Additionally, several authors have reported bigger IC_50_ values for non-irradiated TiNPs compared to the IC_50_ that we obtained with the UV-irradiated mTiNP treatment. As an example, Venkatappa et al. [66] identified that the IC_50_ value of non-irradiated TiNPs was 120 μg/mL against the breast cancer MCF-7 cell line, while Chahardoli et al. [67] found an IC_50_ value around 100 μg/mL against the same cell line. More recently, in 2024, a thiopolyurethane/TiNP composite synthesized by El Sadda et al. [68] displayed an IC_50_ of 122.99  ±  4.07 µg/mL against HepG2 cells and 173.58  ±  6.82 µg/mL against MCF-7 cells. However, other reports have published smaller IC_50_ values that even overcome our UV-enriched NPs treatment [69], suggesting that the synthesis procedure of the nanomaterial and type of cell line are factors that have a strong influence on the variation of this inhibition marker.

It is worth mentioning that NP size is another factor that influences the cytotoxic effect of TiNPs. As a matter of fact, compelling evidence indicates that TiNPs with very small sizes tend to be more cytotoxic [70,71,72]. Particularly, Venkatasubbu et al. [70] obtained an IC_50_ of 26.77 μg/mL for 3 nm non-irradiated TiNPs in HepG2 cells, increasing to 31.48 μg/mL for 4 nm, 49.88 μg/mL for 5 nm, 60.23 μg/mL for 6 nm, and 61.25 μg/mL for 7 nm. These observations indicate that the relatively high IC_50_ obtained in this present study could also be associated with the moderate size of the synthesized TiNPs (64–83 nm). Accordingly, it would be highly advisable to test the anti-cancer properties of our TiNPs at smaller sizes. Moreover, further analyses are recommended to determine the maximum treatment concentration that achieves the highest inhibition, as well as to evaluate the cytotoxic effects in a time-dependent manner.

### 3.3. miRNA Expression After mTiNP Treatment

**Figure 5:**
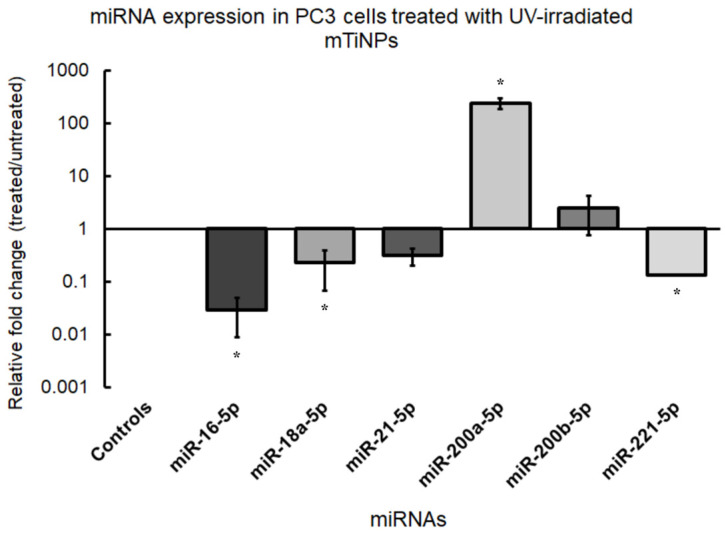
miRNA expression profile of PC3 cells treated with UV-irradiated mTiNPs. The analysis was performed via qPCR on total RNA samples isolated from PC3 cells that had been previously subjected to UV-irradiated mTiNPs (48.96 µg mL^−1^) for 24 h. The U6 was used as the endogenous control for normalization. Each bar graph represents the mean value of the relative fold changes ± the standard error of the biological replicates (* *p*-value < 0.05).

This investigation aimed to explore the differential expression of miR-16-5p, miR-18a-5p, miR-200a-5p, miR-200b-5p, and miR-221-5p in PC3 cells treated with UV-activated mTiNPs, compared to an untreated control group. According to the data obtained from the qPCR experiments, miR-200a-5p and miR-200b-5p were found to be upregulated, while miR-16-5p, miR-18a-5p, miR-21-5p, and miR-221-5p were downregulated, both evaluated in the treated PC3 cells group (Figure 5). The upregulation of miR-200a-5p following treatment was aligned with its established role as a TS in PCa. For instance, Barron et al. [73] demonstrated that the transient overexpression of miR-200a reduced cell proliferation in PCa cell lines, highlighting its antiproliferative effects. Complementing this finding, Guan et al. [74] identified miR-200a as a suppressor of growth and metastasis by targeting Bromodomain-Containing Protein 4 (*BRD4*), a key regulator of androgen receptor signaling that promotes apoptosis. These findings suggest that the anticancer effect of the UV-irradiated mTiNPs could be associated with the activation of miR-200a-5p, as it disrupts tumor cell proliferation and progression through pathways such as BRD4-mediated androgen receptor signaling.

The upregulation of miR-200b-5p after the treatment coincided with its recognized role as a TS in PCa. In this regard, Williams et al. [75] showed that miR-200b overexpression reduced tumor cell proliferation, angiogenesis, and spontaneous metastasis by reversing epithelial-to-mesenchymal transition (EMT). Moreover, Yu et al. [76] demonstrated its ability to suppress proliferation, migration, and stemness in PCa cells by targeting the oncogene *BMI-1*. These findings suggest that the increased levels of miR-200b-5p, induced by the exposure to mTiNPs, may contribute to the inhibition of EMT and tumor cell stemness, thereby reducing cancer progression and metastatic capacity.

On the other hand, the downregulation of miR-18a suggested a potential suppression of oncogenic pathways mediated by the miR-17-92 cluster, to which this miRNA belongs. Zhou et al. [77] observed that the overexpression of this cluster in androgen-independent PCa cells promoted proliferation, migration, and invasion by activating the AKT and ERK1/2 signaling pathways, and, at the same time, it impaired apoptotic mechanisms. Specifically, the miR-17-92 cluster has been found to enhance chemo-resistance by targeting inhibitors of AKT signaling, thereby reinforcing tumor survival. The downregulation of miR-18a observed in our experiments likely mitigated these oncogenic effects, restored apoptotic signaling, and reduced the aggressive behavior of cancer cells. The downregulation of miR-18a was also coherent with the fact that the suppression of the miR-17-92 cluster contributed to autophagy induction via the derepression of the autophagy-related gene *ATG7* [78].

Furthermore, miR-21-5p was found to be downregulated, indicating a possible consequence of the treatment, given that miR-21-5p is typically overexpressed in PCa. In fact, miR-21 is widely recognized as an oncomiR due to its capability to silence TS genes. As an example, Arisan et al. [79] showed that miR-21 promoted EMT and cellular invasiveness through its association with the Wnt-11 signaling pathway. Similarly, Angel et al. [80] linked miR-21 to hypoxia. They also showed that it promoted tumor progression by downregulating the TS *RHOB*, thereby increasing migration and colony formation. The observed downregulation of miR-21-5p in our treated cells suggests that the mTiNPs may effectively disrupt its expression, potentially counteracting its oncogenic activity.

MiR-221-5p has been shown to enhance the proliferation, migration, and invasion of PCa cells by targeting critical pathways and genes. Fortunately, it displayed underexpressed levels in our treated samples. Mercatelli et al. [81] reported that miR-221/222 downregulated p27, a key cell cycle regulator, leading to enhanced tumorigenicity. Similarly, Shao et al. [82] provide evidence that miR-221-5p directly targeted *SOCS1*, thereby promoting EMT. This mechanism facilitated tumor cell proliferation and migration. Remarkably, deletion of miR-221 in PC3 cells resulted in reduced proliferation, invasion, and motility, alongside increased adhesion and alterations in EMT markers [83]. Accordingly, the downregulation of miR-221-5p, detected in our UV-irradiated TiNP-treated samples, could be related to the inhibition of these oncogenic pathways.

Among the analyzed miRNAs, miR-16-5p was the only one displaying a normal expression pattern in PCa, as it was downregulated in the treated samples. This observation aligns with previous findings, which have consistently reported that the TS miR-16-5p is downregulated in different types of cancer, including PCa [50]. Wang et al. [84] demonstrated that the overexpression of miR-16-5p inhibited PC3 cell proliferation, induced apoptosis, and modulated the cell cycle by downregulating AKT3. Additionally, Ghaffari et al. [85] reported that miR-15a delivery decreased *BCL-2* expression and enhanced cell death in PC3 cells. The findings of our investigation, considering the results of previous reports, suggest that the downregulation of miR-16-5p likely reflected its basal expression pattern in PCa and that it is plausible that the mTiNP treatment might not have affected the expression of this miRNA. Therefore, the anti-cancer effect of UV-irradiated mTiNPs may be principally associated, at least, with miR-18a-5p, miR-21-5p, miR-200a-5p, miR-200b-5p, and miR-221-5p.

### 3.4. Differential Expression of PCa-Related Genes After mTiNP Treatment

**Figure 6:**
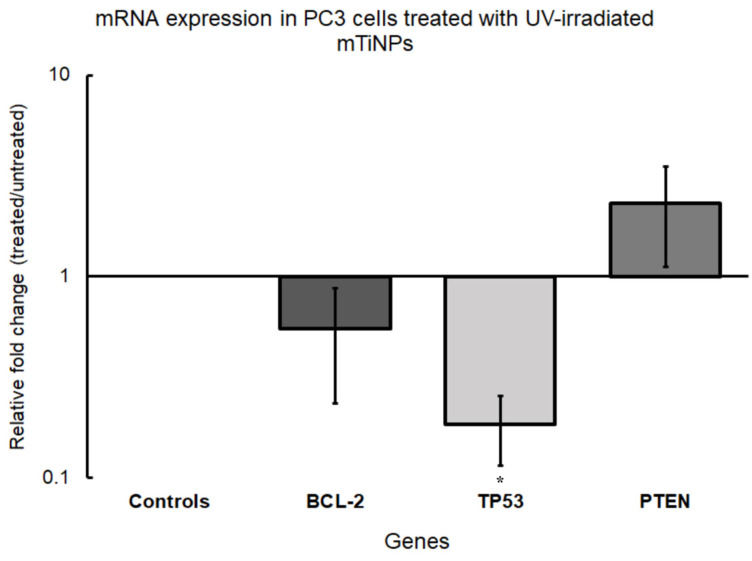
qPCR-based target gene expression in PC3 cells treated with UV-irradiated mTiNPs. The bars represent the mean value of the relative fold changes ± the standard error of the biological replicates (* *p*-value < 0.05).

The downregulation of *BCL-2* observed in the treated samples (Figure 6) reflected the inhibition of this anti-apoptotic protein, which is commonly overexpressed in PCa. *BCL-2* expression has been shown to be transcriptionally regulated by multiple pathways, including the TGF-β/KLF5 signaling axis, which enhances chemoresistance [86]. Notably, Ruiz de Porras et al. [87] identified a marked attenuation of the CXCR2/BCL-2 axis, correlating its downregulation with platinum-based therapies. While miR-16-5p downregulation might suggest increased *BCL-2* expression, it is important to note that miR-16-5p is not the only miRNA that can target this gene, as miR-15a also targets *BCL-2* [85]. The observed downregulation of *BCL-2* in our study may result from the combined effects of multiple miRNAs modulating its expression or even from other regulatory mechanisms of gene expression, which highlights the complexity of the anti-cancer activity of mTiNPs.

The upregulation of *PTEN* detected in the treated PC3 cells indicates another possible antitumor pathway activated by UV-irradiated mTiNPs, as this TS gene plays a central role in regulating cell growth, migration, and invasion by inhibiting the AKT/ERK pathway [88]. Additionally, miR-92a, whose inhibition has been associated with increased *PTEN* expression and reduced phosphorylation of PI3K and AKT, may also play a role in this therapeutic effect [89]. Furthermore, miR-148a and miR-152, which have been reported to suppress *PTEN* in metastatic PCa [90], and miR-1297, which directly targets *PTEN* and promotes tumor progression [88], might also be involved in the therapeutic activity. These findings highlight the critical role of *PTEN* as a therapeutic target in PCa.

**Figure 7:**
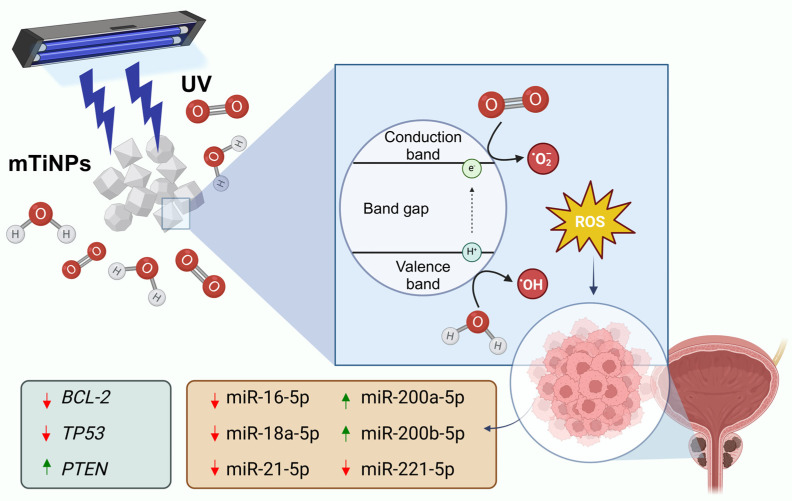
Schematic illustration of the chemical reactions mTiNPs undergo when excited with UV light and the change in miRNA and associated gene expression when in contact with PCa cells. mTiNPs, immersed in an aqueous solution containing water and oxygen molecules, are irradiated with UV light, causing the electrons and holes to transition to the conduction band, generating an electronic shift that allows the generation of reactive oxygen species within PCa cells, causing a differential expression of the indicated miRNAs and associated target genes (created with a licensed version of Biorender).

Finally, the lack of significant upregulation of *TP53* may be explained by the fact that the therapeutic effects of these NPs are independent of p53-mediated pathways. While *TP53* plays a crucial role in cell cycle regulation, apoptosis, and DNA repair [91], the cytotoxicity induced by TiNPs could occur through alternative mechanisms. In this context, Nica et al. [92] showed that TiNPs can trigger apoptosis via pathways that bypass p53. Moreover, as previously mentioned, TiNPs have been more closely related to inducing oxidative stress [34], mitochondrial toxicity [93], or endoplasmic reticulum stress-mediated apoptosis [94], rather than directly depending on p53 activity. This highlights the potential therapeutic applications of UV-irradiated TiNPs in cancers where p53 function is compromised. A potential schematic illustration of the chemical reactions mTiNPs undergo when excited with UV light, along with the differential miRNAs and target gene expression when interacting with PCa cells, is shown in Figure 7.

## 4. Conclusions

Mesoporous TiNPs irradiated by UV light exhibited significantly enhanced cytotoxic activity against PC3 cells, in contrast to non-irradiated mTiNPs, confirming the photocatalytic property of this material. Further, differential miRNA expression analyses revealed that the TS miR-200a-5p and miR-200b-5p were upregulated, while oncogenic miR-21-5p, miR-221-5p, and miR-18a-5p were downregulated after UV-irradiated mTiNPs treatment application. In addition, the anti-apoptotic protein *BCL-2* had a diminished expression, while the TS gene *PTEN* became upregulated. More importantly, the significantly downregulated expression of *TP53* might be confirming apoptosis activation triggered by oxidative stress, known to be induced by the photoactivated mTiNPs. Overall, mTiNPs showed solid potential to become a promising therapeutic agent against PCa. However, additional studies are encouraged to explore the time-dependent dynamics of miRNA and associated gene expression, as well as the extent of cytotoxicity, to assess how longer exposure to UV-irradiated TiNPs influences these factors.

## 5. Future Perspectives

Despite the promising results obtained in this study, further research, along with the integration of multidisciplinary techniques, is necessary to fully evaluate and adapt the application of UV-irradiated mTiNPs for clinical use. Since our observations suggest that treatment with UV-activated mTiNPs could also affect healthy cells, one of the main concerns that should be addressed in forthcoming studies is the targeted delivery of the mTiNPs into PCa cells. In this context, mTiNPs can be functionalized with specific ligands that are readily recognized by PCa cells, such as molecules targeting the prostate-specific membrane antigen (PSMA). In fact, Ngen et al. [95] performed a study in PSMA(+) PC3 PIP tumor-bearing mice, in which they demonstrated that the delivery of PSMA-targeted magnetic nanoparticles (MNPs) was improved by increasing vascular permeability in PSMA(+) PCa tumors through the application of PSMA-targeted photodynamic therapy. Similarly, Sun et al. [96] showed that PSMA-targeted lipid nanoparticles loaded with doxorubicin and tanshinone enhanced the delivery of these drugs into PCa cells, therefore inhibiting tumor growth. Another plausible molecule that could be harnessed for the functionalization of mTiNPs for PCa treatment is transferrin, as the increased expression of transferrin receptors in PCa tissues is well-documented [97]. Remarkably, it has been reported that transferrin-conjugated solid lipid nanoparticles loaded with curcumin facilitated tumor regression in mice bearing PCa tumors [98].

Another significant limitation of the current treatment with UV-irradiated mTiNPs is that UV radiation cannot penetrate into internal tissues and organs such as the prostate. Therefore, it is necessary to develop alternative strategies that enable the photoactivation of mTiNPs within the body without relying on an external irradiation source. As an example, Kotagiri et al. [99] discovered that by utilizing Cerenkov radiation emitted by radionuclides, it is possible to photoactivate TiNPs in vivo. Their approach involved administering transferrin-coated TiNPs along with clinically approved radionuclides (e.g., ^64^Cu and ^18^F) in mice, leading to their colocalization within HT1080 tumors. Consequently, TiNPs were activated by the radiation emitted from the radionuclides, leading to either complete tumor remission or a substantial extension in the median survival of the treated animals. A similar approach was executed by Duan et al. [100], who proved that a combination of dextran-modified TiNPs and ^68^Ga-labeled bovine serum albumin inhibited tumor growth in tumor-bearing mice as a result of the Cerenkov-induced photodynamic activation of TiNPs mediated by ^68^Ga.

Even though this present study did not explore the causes of cell death associated with the UV-activated TiNP treatment, the primary mechanism of cell death mediated by stimulated TiNPs is associated with the generation of intracellular ROS, which causes mitochondrial damage, inhibits ATP synthesis, and triggers apoptosis in cancer cells. Since these effects have been well-documented in numerous scientific reports [27,34,93,101,102,103,104], the main objective of the current work was to analyze how the expression of a set of oncomiRs, TS miRNAs, and associated genes is altered under treatment with UV-irradiated mTiNPs in order to offer a different perspective on the impact of photodynamic therapy on miRNAs and/or target gene expression. This approach also opens the possibility for potential designs of mTiNPs loaded with miRNA-based drugs to enhance the therapeutic effect of our proposal. However, future research must validate whether our mTiNPs also induce cell death in PC3 cells through the aforementioned pathways.

Overall, the subsequent steps in the advancement of the proposed UV-activated mTiNP-based therapy against PCa should focus on the use of mTiNPs functionalized with PCa-cell specific ligands, such as PSMA-targeted molecules or transferrin, and co-formulated with clinically approved radionuclides. Then, these nanoformulations should be rigorously evaluated in PCa animal models to thoroughly assess and validate their therapeutic efficacy, ultimately facilitating their translation into clinical applications.

## Figures and Tables

**Table 1:** Primer sequences used in the qPCR reactions.

miRNA/Gene Name	Forward Primer (5′-3′)	Reverse Primer (5′-3′)
miR-16-5p	TAGCAGCACGTAAATATTGGCG	mRQ 3′ primer (proprietary)
miR-18a-5p	TAAGGTGCATCTAGTGCAGATAG	mRQ 3′ primer (proprietary)
miR-21-5p	TAGCTTATCAGACTGATGTTGA	mRQ 3′ primer (proprietary)
miR-200a-5p	CATCTTACCGGACAGTGCTGGA	mRQ 3′ primer (proprietary)
miR-200b-5p	CATCTTACTGGGCAGCATTGGA	mRQ 3′ primer (proprietary)
miR-221-5p	ACCTGGCATACAATGTAGATTT	mRQ 3′ primer (proprietary)
*BCL-2*	GATGGGATCGTTGCCTTATGC	CTTGGCATGAGATGCAGGA
*TP53*	ACCTATGGAAACTACTTCCTG	ACCATTGTTCAATATCGTCC
*PTEN*	AGTCAGAGGCGCTATGTGT	CGTGTGGGTCCTGAATTGGA
*U6*	GGAACGATACAGAGAAGATTAGC	TGGAACGCTTCACGAATTTGCG

## Data Availability

The data that support the findings of this study are available from the corresponding author upon reasonable request.
